# A Comparative Qualitative Study of Misconceptions Associated with Contraceptive Use in Southern and Northern Ghana

**DOI:** 10.3389/fpubh.2014.00137

**Published:** 2014-09-05

**Authors:** Philip B. Adongo, Philip T.-N. Tabong, Thomas B. Azongo, James F. Phillips, Mallory C. Sheff, Allison E. Stone, Placide Tapsoba

**Affiliations:** ^1^Department of Social and Behavioral Sciences, School of Public Health, University of Ghana, Accra, Ghana; ^2^School of Medicine and Health Sciences, University for Development Studies, Tamale, Ghana; ^3^Department of Population and Family Health, Mailman School of Public Health, Columbia University in the City of New York, New York, NY, USA; ^4^The Population Council, Accra, Ghana

**Keywords:** misconceptions, contraceptives, family planning, unmet need, Ghana

## Abstract

Evidence from Ghana consistently shows that unmet need for contraception is pervasive with many possible causes, yet how these may differ by cultural zone remains poorly understood. This qualitative study was designed to elicit information on the nature and form of misconceptions associated with contraceptive use among northern and southern Ghanaians. Twenty-two focus group discussions (FGDs) with married community members were carried out. Community health officers, community health volunteers, and health care managers were also interviewed using a semi-structured interview guide. FGDs and in-depth interviews were recorded digitally, transcribed verbatim, and analyzed using QSR Nvivo 10 to compare contraceptive misconceptions in northern and southern Ghana. Results indicate that misconceptions associated with the use of contraceptives were widespread but similar in both settings. Contraceptives were perceived to predispose women to both primary and secondary infertility, uterine fibroids, and cancers. As regular menstrual flow was believed to prevent uterine fibroids, contraceptive use-related amenorrhea was thought to render acceptors vulnerable to uterine fibroids as well as cervical and breast cancers. Contraceptive acceptors were stigmatized and ridiculed as promiscuous. Among northern respondents, condom use was generally perceived to inhibit erection and therefore capable of inducing male impotence, while in southern Ghana, condom use was believed to reduce sensation and sexual gratification. The study indicates that misconceptions associated with contraceptive use are widespread in both regions. Moreover, despite profound social and contextual differences that distinguish northern and southern Ghanaians, prevailing fears and misconceptions are shared by respondents from both settings. Findings attest to the need for improved communication to provide accurate information for dispelling these misconceptions.

## Introduction

Estimates suggest that sexual and reproductive conditions account for 18.4% of the global burden of disease, of which 32.0% is among women aged 15–44 years (1). Contraception and family planning are integral components of reproductive health, and have demonstrated positive effects on women’s health. Moreover, family planning promotion has the potential to reduce poverty, maternal and child mortality, high risk pregnancy, and abortion (2). Because of this importance, universal access to reproductive health services is identified as one of the targets of the United Nations Millennium Development Goals (3). The World Health Organization has acknowledged the priority need for family planning services that enables couples to implement preferences to space or limit childbearing. Yet research in many settings has demonstrated that couples often eschew family planning out of concern for its detrimental effects. Indeed, as is the case with all pharmaceuticals, all contraceptive methods have one or more known side-effects.

Despite these known side-effects, several other complex reasons are cited by women when interviewed about perceptions of risk and reasons for non-use (4). Of these factors, some have no obvious link to side-effects, such as lack of concern over the possibility of pregnancy, perceived invulnerability to pregnancy, and forgetfulness (5). Other factors are correlates rather than direct causes, such as low socio-economic status, low educational attainment, or cultural barriers to contraceptive use (6). Poor access, cost, or clinical restrictions are also cited as barriers to this use (7). However, misconceptions about family planning and the effects of contraceptives on women’s health, future fertility, and birth outcomes are further mentioned as reasons for non-use (8).

Contraceptive use in West Africa has been lower than levels reported in East and Southern Africa, despite longstanding attention from foreign aid programs and national policies (9). In 2004, Ghana projected that it would achieve a contraceptive prevalence rate (CPR) of 28% by 2010 and of 50% by 2020. The attainment of these goals was recognized as integral to the country’s economic development national strategy as outlined in the Vision 2020 Plan of Action (10). However, records indicate that Ghana’s current contraceptive acceptance rate is 23%, five percentage points below the expected projection. Several factors may be responsible for Ghana’s inability to achieve this target, notably barriers in contraceptive use that have often been cited as one of the reasons impeding the achievement of fertility regulations (11). Understanding these barriers is important for providing programing guidance on the provision of family planning services. This qualitative descriptive study was therefore designed to elicit from community members misconceptions associated with the use of contraceptives, and compare and contrast these between southern and northern Ghana. Knowledge of these perceptions in different regions is important for the design of programs targeting false impressions.

## Materials and Methods

### Study areas

The study was conducted in two districts each in northern and southern Ghana. Sefwi Bibiani-Ahwiaso Bekwai (SBAB) district in Western region and Komenda-Edina-Eguafo-Abrem (KEEA) municipal area in the Central region comprised localities of the southern zone, whereas Kassena-Nankana east (KNE) and west (KNW) districts in the Upper East Region of Ghana comprised the localities in the northern Ghana. The 2010 Population and Housing Census reported the populations for the SBAB and KEEA as 123,272 and 144,705, respectively. The same report indicated that the population of KNE and KNW was 109,944 and 70,667, respectively (8). The Ghana Demographic and Health survey further noted that the contraceptive prevalence for modern contraceptive methods among married women between the ages of 15 and 49 years was 14% for the Upper East Region, 17% in the Central Region, and 13% in the Western Region (12). KNE and KNW were selected because of an ongoing community health family planning model, which was being piloted to determine the impact of the program on fertility in northern Ghana. SBAB and KEEA were also selected because the districts received support from a USAID grant through the Population Council of Ghana to implement Community-based Health Planning and Services (CHPS). The CHPS strategy, among other objectives, aims to increase community members’ access to reproductive health and family planning.

### Study design

This study was a descriptive qualitative study using in-depth interviews, focus group discussions (FGDs), and expert opinions. These strategies were employed to elicit in-depth information on misconceptions regarding the use of contraceptives in the selected districts and understand the reasons for these misconceptions.

### Data collection

Ghana Health Service Ethics Review Committee approved the protocol for the study. Participants in the study gave either written or verbal consent as an indication for their willingness to participate. Verbal consents were recorded digitally and those who gave verbal consent were asked to recommend an independent person to serve as witness to the process. Participants who gave written consent were asked to either append their signature or thumbprint on informed consent forms as an indication of their agreement to take part in the study. In order to ensure confidentiality and anonymity, codes were used to identify participants in both in-depth interviews and FGDs. Names and locations of communities were also kept confidential.

Semi-structured in-depth interview guides were designed by the researchers and used to collect the data. These interview guides were designed in English and translated into Akan, Kassim, and Nankana by language experts using back-to-back strategy. In back-to-back strategy, independent language experts first translated the primary instruments in English into the various local languages. Another group of language experts proficient in both English and the local languages retranslated the versions from the local languages back to English. The two versions are then compared to ensure consistency. Inconsistencies were resolved through a discussion between the language experts and an independent referee. Local research assistants were recruited, trained, and deployed to their linguistically relevant communities. Training for interviewers included a combination of classroom work and mock interview exercises.

Twenty-two FGDs were held in northern and southern Ghana; 11 FGDs for male groups, and 11 with female homogenous community members. All participants were married with children. Focus group discussants consisted of 6–8 people seated in a semi-circle with the moderator and note-taker sitting in front of the discussants. During FGDs, each participant was given the opportunity to give their contribution on a particular question before proceeding to another question. In many instances, there was consensus in responses. The sampling of the respondents was carried out to ensure that both urban and rural residents were represented in all study areas. In addition, community health officers, volunteers, and health managers were purposefully recruited and interviewed using a semi-structured interview guide. Both IDIs and FGD lasted for between 30 and 90 min and were conducted within communities.

### Data processing and analysis

Both FGDs and IDIs were audiotaped using a digital audio-recorder, and complemented with written interview notes on paper. The study coordinators crosschecked all the data received for completeness and accuracy on a daily basis. Content analysis was used to analyze the qualitative data based on emerging themes and sub-themes in line with the study objectives. The researchers designed an initial codebook, which was accepted by all researchers. Based on the codebook, coding of data was carried out using QSR Nvivo 10^©^, a computer program for analyzing qualitative data sets. Trend analysis of the FGDs and IDIs for each topic was used to identify the major issues for each of the study themes and sub-themes. The trend analysis was also employed to facilitate comparison of the views of participants within and among the different study areas. Descriptive narratives supported by illustrative quotes are used in results.

## Results

### Contraceptive use and changes in weight

The majority of women, who use contraceptives, do not gain or lose weight; however, this was a main misconception found in this study. Weight changes occur naturally in life but because these changes in weight are so common, many women misconstrue this and attribute these changes to the use of contraceptives. Both weight gain and loss were mentioned in northern and southern Ghana; weight loss was more pronounced in southern Ghana, while respondents in northern Ghana were more inclined to attribute the use of contraceptives to weight gain.

My little sister went to do the implant and she changed drastically, that is at first, she was fat but after doing the family planning she grew lean. Because of what happened, my mother advised her to stop the FP and as soon as she stopped using the FP, she regained her weight back

— (woman, FGD, southern Ghana)

Many people say it brings a lot of problems to them especially when it is incompatible with their blood and body. Some also say that it makes them put on a lot of weight

— (woman, IDI, northern Ghana)

Both weight gain and loss were perceived as undesirable for women as it had the tendency to cause marital instability.

They think that if you do family planning and grow very fat your husband will not like you again and will go out for another woman

— (CHO, IDI, northern Ghana)

### Contraceptive use and cancers

Associating contraceptive use and many forms of cancers in women emerged as a well-entrenched theme. In both settings, community members believed that contraceptive use predisposed women to cancers. In southern Ghana, the use of contraceptives allegedly predisposed women to uterine fibroids (myomas).

…. I know another lady who did it (FP) and stopped menstruating, later on it developed into fibroid and ended up in surgery

— (man, FGD, southern Ghana)

Yes, they have misconceptions about family planning; when you do it (FP) blood will clot in your womb, so that it would turn into fibroid … they believe that a Jadelle that is implanted, can penetrate through your heart and go somewhere that can result in death. And the IUCD, some also think that when you insert it, IUCD would penetrate into your uterus and cause cancer

— (CHO, IDI, southern Ghana)

Apart from the perception that the use of contraceptives could predispose a woman to uterine fibroid in southern Ghana, in northern Ghana, the use of contraceptive was further associated with other type of cancers, such as cervical and breast cancers.

The community members perceive that when a woman uses IUD with time it moves from the original place to another part of the body to cause cancers in various parts of your body such as the uterus and breast

— (CHO, IDI, northern Ghana)

Some respondents even attributed the high incidence of cancers in present times to the use of contraceptives. To some, ancestors did not get cancers because they never used contraceptives. Although no scientific evidence supports the claim, respondents in northern Ghana perceived that the incidence of cancers was higher in southern Ghana as a result of higher contraceptive prevalence in those regions.

Our ancestors were not getting cancers because they did not do family planning

— (man, IDI, northern Ghana)

So you see when our women go to the south, because they do FP there, they end up getting [more] cancers than the women here in the north

— (man, IDI, northern Ghana)

### Traditional values

Traditional values still play a major role in contraceptive uptake and adherence, especially in northern Ghana, and emerged as a well-entrenched theme in northern Ghana. Respondents were asked to describe the norms and practices that might affect the uptake of contraceptive services in the community to determine how project interventions can be successful. The traditional belief that contraceptive use is synonymous to abortion emerged as a drawback to contraceptive acceptance, traditional myths more pronounced in northern Ghana.

It is believed that traditional people should not use contraceptives. You need to give birth to the number of children the gods have given to you

— (man, IDI, northern Ghana)

Another area of concern was the desire to beget more children, especially in northern Ghana. Respondents in that region alluded to changing perception of high fertility rates as ideal and acceptable in the community; yet in northern Ghana, the belief that using contraceptives could offend their gods and ancestors prevents many couples from accepting contraceptives.

Some people hold the belief that when you use contraceptives you will offend the gods but this perception is gradually fading off

— (man, IDI, northern Ghana)

### Perception of contraceptive use and illness

Participants in both southern and northern Ghana cited the expulsion or shifts to other parts of the body of Intrauterine Contraceptive Device (IUCD) or implants as cause for concern. Community members believe that implants could dislodge and go missing in the body through the blood stream, causing discomfort.

We have heard about people complaining that they are not feeling well or they frequently fall sick when using contraceptives

— (woman, FGD, southern Ghana)

I personally did FP, I went for one month injection and I was continuously getting sick, I really suffered during that time so I stopped. I am not someone who usually gets sick but it was not so during the time I was using the FP method

— (woman, FGD, southern Ghana)

Some people say that when they use the family planning, they feel dizzy

— (woman, FGD, southern Ghana)

Like the way my sister is saying, for me what I heard is that when some people do it they bleed and feel dizzy. For me, I have not done some before but these are the kind of things that I hear

— (woman, FGD, southern Ghana)

In the community people are speculating about the side effects of some of the FP products, some say they get excruciating pain, others say they suffer in their heart and also dizziness which could let you faint, others also say they often suffer from high blood pressure when they use contraceptives

— (CHO, IDI, northern Ghana)

Feelings of dizziness following the use of contraceptive could be a genuine complaint, which will require further investigation and counseling. In the absence of professional guidance, these adverse effects may be amplified in the community as an effect all contraceptive users experience. Closely related to this is the perception of vomiting after sex, which is attributable to the use of contraceptives among communities in northern Ghana.

Others say that they get stomach related problems. Some say that when they practice FP and have sex with a man, they become nauseated and vomit

— (CHO, IDI, northern Ghana)

### Contraceptive use and infertility

There is no scientific evidence linking the use of contraceptive to infertility. However, this is a widely held perception that emerged as a well-entrenched theme in both southern and northern settings. Communities in northern Ghana generally believed that contraceptive use was inappropriate for people who had not given birth, and that their use could lead to permanent childlessness due to loss of fecundity. Although this association did not emerge as a prominent theme in southern Ghana, linking contraceptive use to a secondary cause of infertility was mentioned in both southern and northern Ghana.

Some also say if you do it (FP) for a long time you will not give birth but if you do it for a short period and you want to get pregnant you really suffer

— (woman, FGD, southern Ghana)

Yes, they (misconceptions) are very common here because most of them have in mind that when you do it (FP), you will not give birth again. With depo, when you do it, you do not bleed (menstruate), because you do not bleed they think the blood accumulates in your womb and will give you problems in future making it impossible for you have child

— (man, IDI, northern Ghana)

With the IUCD, you know that one passes through your vagina, some people think that it might shift to your womb and that way you will not be able to give birth again

— (CHO, IDI, northern Ghana)

With the perception of the association between contraceptive use and primary infertility in northern Ghana, contraceptives were viewed as unsuitable for women, who have never given birth if they were concerned about having children in future.

… if you have not given birth before, you do not have to use contraceptive as you will not be able to give birth in future

— (CHO, IDI, northern Ghana)

FP is not good for people who have not given birth before as they may not be able to give birth in future

— (man, IDI, northern Ghana)

### Perception of the effects of contraceptives on physical and intellectual ability of children

Evidence shows that contraceptive use does not cause birth defects, nor will it harm the fetus if a woman becomes pregnant while taking contraceptives. However, northern community members believed that the use of contraceptives is associated with birth defects. According to these respondents, women who have used contraceptives are more likely to give birth to children with defects or intellectually impaired.

Some people believe that if you use contraceptive, you will give birth to an abnormal child

— (man, IDI, northern Ghana)

Apart from the bleeding, others are also saying that when you are on it (FP) for a long period, when you become pregnant the child will not be intelligent, the child will not be that good in school and the child will always fall sick

— (CHO, IDI, northern Ghana)

In this community, they believe that people that use contraceptive give birth to children that are intelligent

— (man, IDI, northern Ghana)

However, linking contraceptive use to birth effects was not common in southern Ghana as it was neither mentioned in IDIs nor FGDs.

### Contraceptives use and promiscuity

Associating the use of contraceptives to a promiscuous lifestyle emerged as a major theme, more predominant in southern than in northern Ghana. The general belief in southern Ghana is that married women who want to engage in extramarital affairs employ contraceptives as a strategy to prevent unplanned pregnancies.

Women who want to cheat on their husbands (engage in extramarital affairs) are the people who use contraceptives, so that when their husbands are not around, they can be sleeping with other men after all they cannot become pregnant for the husband to detect any extramarital affairs

— (man, FGD, southern Ghana)

…. Family planning makes women to go out to have sex with other men, so they would not allow their wives to do it

— (CHO, IDI, northern Ghana)

With this perception, men resist any attempt of their wife to use contraceptives as this may indicate support for your wife to engage in extramarital affairs.

### Perception of condom use and penile erection

The effect of condom use on penile erection has no anchor in science, yet respondents in northern Ghana believed that condoms prevent them from erecting and sustaining an erection. The use of condoms, respondents believed, could lead to impotency, especially when used frequently during sex and over a long duration.

They say that when they use the condom, their penis is not able to erect

— (CHO, IDI, northern Ghana)

As for the use of condom, we do not have to talk about it, it is like eating a toffee with the wrapper … it is even difficult to sustain an erection when you are using condom

— (man, IDI, northern Ghana)

Most of men in this community do not like using condom because they say it is always too tight on their penis making it difficult for them to erect

— (CHO, IDI, northern Ghana)

However, in southern Ghana, condom use was believed to reduce sensation for the man, thereby reducing sexual pleasure.

As for condom, let not talk about at all, you will not enjoy sex if you use condom

— (man, FGD, southern Ghana)

### Vasectomy, sexual, and physical weakness

Vasectomy has no effect on both the physical and sexual ability of men. However, this emerged as a misconception in southern Ghana. For respondents there, vasectomy was capable of making a man both physically and sexually weak. The physical weakness, respondents believed, could make the man less productive and therefore incapable of meeting the socio-economic needs of the family. This perception was firmly entrenched in the minds of both men and women. Closely related to the perceived physical weakness is the perception of a reduction in the man’s sexual ability. Many respondents believed this could lead to marital instability as it could encourage women to engage in extramarital affairs in order to the get the sexual gratification the man could no longer provide.

Vasectomy will make the man weak and will not be able to perform very well his physical and sexual duties in the family

— (woman, FGD, southern Ghana)

It is the women who do not allow their husbands to do it (vasectomy) because they believe that it (vasectomy) will make the man both physically and sexually weak

— (Health Manager, IDI, southern Ghana)

Knowledge levels on vasectomy were generally low among respondents in northern Ghana, and the procedure was perceived as synonymous to castration in both settings and therefore inappropriate for a man.

## Discussion

This descriptive qualitative study was designed to explore the misconceptions associated with the use of contraceptives, and to compare these similarities and differences in northern and southern Ghana. The study delineates that misconceptions associated with contraceptive use are still widespread among community members in both settings. These misconceptions are impediments to the advocacy to increase contraceptive prevalence, and in extension, impediments to combating unwanted pregnancy and unsafe abortion (13). However, the study also reveals the need for innovative ways to dispel misconceptions in the community. The majority of these misconceptions are spread by community members and are transferred to other geographical areas by people without in-depth knowledge of the subject matter. Therefore, there is need for an increase in human resources with requisite knowledge on contraceptives. In the absence of qualified counseling and advice on the use of different contraceptive methods, clients are compelled to rely on friends and family members for information, which in many cases may be clouded with misconceptions.

The fear and misconstructions in both northern and southern Ghana appear to be linked to undesirable outcomes in the use of contraceptives by previous users. One is related to the inability to fulfill the reproductive role, such are begetting a child or delays in return to fertility when contraceptives are stopped. This is problematic given the high value that is placed on children in the community, and even more pronounced in northern Ghana where a man’s wealth is customarily measured by the number of biological children he has. These, entwined with severe social consequences of emotional strain in a relationship, fear of abandonment, and general community stigma, fuel misconceptions creating an uncertain environment for future users. Early sexual and reproductive health education may be appropriate to ensure that individuals acquire knowledge early enough to distil between misconceptions and adverse reactions. Couples-based counseling is also highly recommended because of the role men play in the reproductive health decision-making process.

The perception of the association between contraceptive use and serious complications such as cancers or birth defects would not only make it difficult for a woman to fulfill her reproductive role in a marriage, but also have perceived financial implications in terms of medical costs. In effect, women who cannot overcome fear of spousal abandonment or neglect in case of method complications may likely opt not to use contraception until their spouse agrees to it. A recent study in southern Ghana revealed that spousal consent was still very relevant in contraceptive uptake among women (11). Programs should therefore be designed to target men to discredit these misconceptions. A man who receives correct information on the use of contraceptives is more likely to positively influence his wife’s use and disabuse the myths.

If the use of the male condom is believed to prevent erection and subsequent impotence, the situation could be used as an opportunity to promote the use of female condoms. Previously launched female condoms did not achieve the desired results because of low patronage (14) leading to the launch of the second-generation female condom. Condom use is one of the key strategies espoused by the National AIDS Control Program (NACP) as a result of its dual protection against pregnancy and sexually transmitted infections (STIs). The notion that condom use inhibits penile erection therefore poses a challenge to this strategy and, more generally, to the control of STIs in Ghana. More education is required on this topic, and reproductive health advocates should act upon this observation to strengthen campaigns in favor of the use of the female condom as an alternative to male condom. With correct and consistent use, the female condom is as effective as other barrier methods and has no known adverse effects or risks (15, 16).

Oral contraceptives offer many non-contraceptive health benefits, including decreased risks of bone loss, benign breast disease, pelvic inflammatory disease, ectopic pregnancy, and rheumatoid arthritis (17). However, these benefits are often overshadowed by the myths this study revealed. Although some studies have reported an association between oral contraceptives and breast cancer, the relative risk has been reported to be very small (18). However, contraceptive use does not predispose women to uterine fibroid. The use of oral contraceptives has instead proven to be effective in preventing endometriosis, ovarian cancers, and colorectal cancer (18).

Associating contraceptive use to women’s promiscuous lifestyle poses another challenge to the push to increase contraceptive uptake. With this perception, many women who are willing to use contraceptives may desist from usage or engage in concealed use. This calls for a restructuring of contraceptive outlets. Institutionalized-based delivery of contraceptive services may be inappropriate for communities who stigmatize their use. The findings of this study also raise questions about continued use of contraceptives, especially for women whose husbands have traveled as there is a propensity to categorize a woman who is using contraceptives in the absence of her husband as promiscuous. An earlier study has documented that women cited the absence of their husbands as reasons for not using contraception because it was considered socially unacceptable for such women to use contraception (19).

## Methodological Considerations

The authors used independent language experts to do the translations from local languages to English. These translations were verified. However, it is possible some of the original words could have lost their meaning. To mitigate this weakness, in determining the themes, emphasis was placed on overarching themes present in the transcripts rather than specific words or phrases used by respondents. In northern Ghana, two districts from the Upper East Region of Ghana were selected; it will therefore be necessary for future research to select districts in other regions of northern Ghana to give a fair representation of the cultural misconceptions present in the North.

## Conclusion

The study indicates that contraceptive misconceptions were widespread among panels of men and women in both northern and southern Ghana. Results attest to the need for improved and intensified provision of accurate information to alleviate fear, anxiety and misconceptions in both southern and northern Ghana. Findings also suggest that common problems prevail, despite profound differences in cultural circumstances. But, while communication is needed to address misconceptions about risks and side effects, the commonality of problems is not necessarily suggestive of common strategic solutions. Practical means of engaging men, reaching women, and improving the climate of knowledge may require markedly different approaches to dispel these barriers. Nonetheless, ubiquitous misconceptions, evident in every focus group and every interview, suggest that implementation research to clarify myths and concerns is urgently needed.

## Author Contributions

Philip Baba Adongo, James F. Phillips, Placide Tapsoba, Allison E. Stone conceived and designed the study: Philip Baba Adongo, Placide Tapsoba, Allison E. Stone, participated in data collection; Philip Baba Adongo, Philip T.-N. Tabong, Thomas B. Azongo, Mallory C. Sheff, did the analysis and writing of the manuscript. All authors read and approved the final manuscript.

## Conflict of Interest Statement

The authors declare that the research was conducted in the absence of any commercial or financial relationships that could be construed as a potential conflict of interest.
